# A novel mode of chromosomal evolution peculiar to filamentous Ascomycete fungi

**DOI:** 10.1186/gb-2011-12-5-r45

**Published:** 2011-05-24

**Authors:** James K Hane, Thierry Rouxel, Barbara J Howlett, Gert HJ Kema, Stephen B Goodwin, Richard P Oliver

**Affiliations:** 1 CSIRO Plant Industry, Centre for Environment and Life Sciences, Private Bag 5, Perth, 6193, Australia; 2 Faculty of Health Sciences, Murdoch University, Perth, 6150, Australia; 3 INRA-Bioger, Avenue Lucien Brétignières, BP 01, Thiverval-Grignon, 78850, France; 4 School of Botany, The University of Melbourne, Melbourne, 3010, Australia; 5 Wageningen UR, Plant Research International, Department of Biointeractions and Plant Health, PO Box 69, Wageningen, 6700 AB, The Netherlands; 6 USDA-ARS, Crop Production and Pest Control Research Unit, Purdue University, 915 West State Street, West Lafayette, IN 47907-2054, USA; 7 Australian Centre for Necrotrophic Fungal Pathogens, Curtin University, Perth, 6845, Australia

## Abstract

**Background:**

Gene loss, inversions, translocations, and other chromosomal rearrangements vary among species, resulting in different rates of structural genome evolution. Major chromosomal rearrangements are rare in most eukaryotes, giving large regions with the same genes in the same order and orientation across species. These regions of macrosynteny have been very useful for locating homologous genes in different species and to guide the assembly of genome sequences. Previous analyses in the fungi have indicated that macrosynteny is rare; instead, comparisons across species show no synteny or only microsyntenic regions encompassing usually five or fewer genes. To test the hypothesis that chromosomal evolution is different in the fungi compared to other eukaryotes, synteny was compared between species of the major fungal taxa.

**Results:**

These analyses identified a novel form of evolution in which genes are conserved within homologous chromosomes, but with randomized orders and orientations. This mode of evolution is designated mesosynteny, to differentiate it from micro- and macrosynteny seen in other organisms. Mesosynteny is an alternative evolutionary pathway very different from macrosyntenic conservation. Surprisingly, mesosynteny was not found in all fungal groups. Instead, mesosynteny appears to be restricted to filamentous Ascomycetes and was most striking between species in the Dothideomycetes.

**Conclusions:**

The existence of mesosynteny between relatively distantly related Ascomycetes could be explained by a high frequency of chromosomal inversions, but translocations must be extremely rare. The mechanism for this phenomenon is not known, but presumably involves generation of frequent inversions during meiosis.

## Background

The evolutionary history of organisms, as revealed by comparisons of genome sequences, is of the greatest biological significance and interest. The current explosion in the number of genome assemblies of species within the same class, order and genus is allowing the whole-genome interrelationships between organisms to be examined in ever greater detail. There is a long history of comparisons of individual orthologous gene sequences and these have revolutionized our understanding of phylogenetic relationships [1]. A more complete understanding of both the mechanism and results of evolution can be obtained by comparing entire genomes [2]. These comparisons have refined the concept of synteny. This term is used loosely by many authors. Originally it was used in cytogenetics to describe two or more loci that are located on the same chromosome. As DNA sequencing and comparative genomics became commonplace, the term synteny acquired the additional property of co-linearity; i.e. the conservation of gene order and orientation. In this study we refer to synteny in the original cytogenetic sense and describe co-linearity as a sub-category of synteny. If orthologs of multiple genes that are co-located in the genome of one organism are co-located in another species, the chromosomes on which the genes reside are said to be syntenic. Synteny can also be quantitative; chromosomes that contain all of the same genes are 100% syntenic.

The process of speciation occurs when two independent populations diverge into reproductively isolated species. Initially the daughter species would have had chromosomes that shared both gene content (synteny) and order (co-linearity). Over evolutionary time, the degree of synteny and co-linearity would be degraded through various processes, including chromosomal duplications, gene losses/gains and chromosomal rearrangements (Additional file 1), until orthologous genes in one species occur randomly in the genome of the other.

The related concepts of synteny and co-linearity have been refined mostly in plants, animals and bacteria. Synteny has been differentiated qualitatively based on the length and completeness of co-linear regions. Macrosynteny describes co-linearity observable at a whole-chromosome scale, involving hundreds or thousands of genes of which a backbone are co-linear. Microsynteny describes co-linearity spanning a small number (for example, two to ten) of successive genes. Comparisons of vertebrate and flowering plant species within taxonomic families often have shown extensive macrosynteny [3-8]. Macrosynteny has been exploited to assist genetic mapping and gene cloning; examples include the use of the *Arabidopsis *genome to find genes in canola [9], and rice/*Brachypodium *synteny to locate genes in wheat and barley [10].

**Figure 1:**
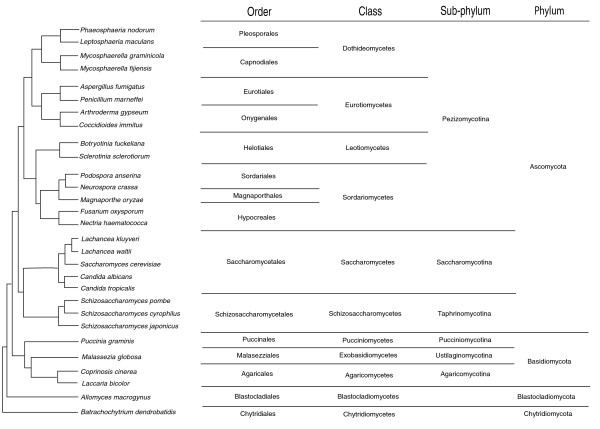
**Cladogram of species used in whole-genome comparisons in this study**. Detailed information on each species is provided in Additional file 3.

Filamentous fungi form an ancient, large and diverse group of organisms. Until the last decade, the phylogenetics of fungi was problematic but the application of techniques based on gene sequence variation has created a stable taxonomy. The ascomycete filamentous fungi are mostly within the sub-phylum Pezizomycotina (Figure 1) [11]. This sub-phylum contains four major classes: Dothideomycetes, Eurotiomycetes, Sordariomycetes and Leotiomycetes. The Dothideomycetes contains more than 20,000 species amongst which are many of the most important plant pathogens worldwide, including those in the genera *Phaeosphaeria*, *Leptosphaeria *and *Mycosphaerella*.

Evolutionary diversity within the filamentous ascomycete fungi is much higher than in flowering plants or vertebrate animals [12]. A number of reasons have been proposed to account for this. Filamentous fungi reflect approximately 400 million years of evolutionary history, comparable to that of the vertebrates but approximately four times longer than that of the flowering plants [13] (Figure 1; Table 1). The generation times of fungi are typically measured in hours or days, whereas plants and animals have generation times of many weeks, years or even decades. Meiosis is a powerful force stabilising chromosomal structure and may occur less commonly in some fungi compared to plants and animals; whilst nearly all filamentous fungi undergo germline asexual reproduction, only a subset have known sexual phases. Furthermore, many filamentous fungi can acquire genetic material by lateral gene transfer, which can increase their rate of evolution [14-16]. All of these factors would tend to reduce or eliminate the extent of synteny between species. It was not surprising, therefore, when initial comparisons between fungal genome sequences failed to find extensive evidence of interspecific macro- or microsynteny [17-22] and, with the exception of the aspergilli, even between species from the same genus [23-25].

**Table 1:** Summary of whole-genome synteny relationships across selected fungal orders

	Sub-phylum	Pezizomycotina								Saccharomycotina	Taphrinomycotina
		
	Class	Dothideomycetes		Eurotiomycetes		Sordariomycetes			Leotiomycetes	Saccharomycetes	Schizosaccharomycetes
					
Class	Order	Capnodiales	Pleosporales	Eurotiales	Onygenales	Hypocreales	Magnaporthales	Sordariales	Helotiales	Saccharomycetales	Schizosaccharomycetales
Dothideomycetes	Capnodiales	Meso/150	Meso	Demeso	Demeso	Demeso	None	Demeso	Demeso	None	None
	Pleosporales	300	Meso/120	Demeso	Demeso	Demeso	None	Demeso	Demeso	None	None
Eurotiomycetes	Eurotiales	370	370	Demacro/< 160	Demacro	None	None	None	Demeso	None	None
	Onygenales	370	370	150	Demacro/< 160	None	None	None	Demeso	None	None
Sordariomycetes	Hypocreales	370	370	370	370	Macro/170	Demeso	Demeso	Demeso	None	None
	Magnaporthales	370	370	370	370	240	NA	Demeso	Demeso	None	None
	Sordariales	370	370	370	370	225	240	Demeso	Demeso	None	None
Leotiomycetes	Helotiales	370	370	370	370	340	340	340	Macro/250	None	None
Saccharomycetes	Saccharomycetales	500	500	500	500	500	500	500	500	Demacro/none/250	None
	Schizosaccharomycetales	650	650	650	650	650	650	650	650	650	Demacro/none/240

Whole-genome synteny, indicated above the diagonal, was classified as either macrosynteny (macro), degraded macrosynteny (demacro), mesosynteny (meso), degraded mesosynteny (demeso) or no synteny (none). 'NA' indicates lack of sufficient data to perform whole-genome comparisons. Time since divergence between orders, previously predicted within the Ascomycetes [38], is indicated below the diagonal in millions of years.

The number of sequenced fungal genomes has increased dramatically since 2008. There is now a sufficient number of sequenced species within each fungal class to begin to assess whole-genome patterns of evolution. In this paper, we have applied a simple dot-plot approach to fungal genome comparisons and observed a striking pattern of chromosome-level evolutionary conservation. This pattern is characterized by the conservation of gene content in chromosomes, without conservation of gene order or orientation; that is, synteny without co-linearity. We propose to call this sequence conservation 'mesosynteny' to distinguish it from micro- and macrosynteny. Mesosynteny appears to be peculiar to the filamentous Ascomycetes (syn. Pezizomycotina), particularly in the class Dothideomycetes. This phenomenon has interesting implications for the study of genome evolution and may have applications in the sequencing and assembly of fungal genomes.

## Results

Dot plots are a well-established method of representing sequence comparisons [26]. Comparison of co-linear genomes (Supplementary Figure S1a in Additional file 1) gives a series of dots that lie on the diagonal (Supplementary Figure S1b in Additional file 1). Random gene loss from either chromosome without major rearrangements (Supplementary Figure S1c, d in Additional file 1) progressively destroys microsynteny but retains macrosynteny. Inversions are visualised on dot plots by diagonal lines with the opposite slope, while translocations are indicated when the genes on a chromosome of one species share syntenic blocks with two or more chromosomes. Conservation of short, contiguous runs of genes, whether on the same or different chromosomes, retains microsynteny but not macrosynteny.

The fungus *Phaeosphaeria *(syn. *Stagonospora*, *Septoria*) *nodorum *is a major pathogen of wheat [27]. It is a member of the class Dothideomycetes (Figure 1), a taxon that includes more than 20,000 species amongst which are many dominant crop pathogens [28]. Its genome, which is believed to comprise 14 to 19 chromosomes [29], was assembled as 107 nuclear scaffolds [21]. Expressed sequence tag and proteomic data have refined the annotations to a set of 12,194 genes [30-32]. Pathogenicity in *P. nodorum *has been linked to the expression of a suite of necrotrophic effectors [33-36] (formerly called host-specific toxins), some of which appear to have been acquired by lateral gene transfer [14,16].

**Figure 2:**
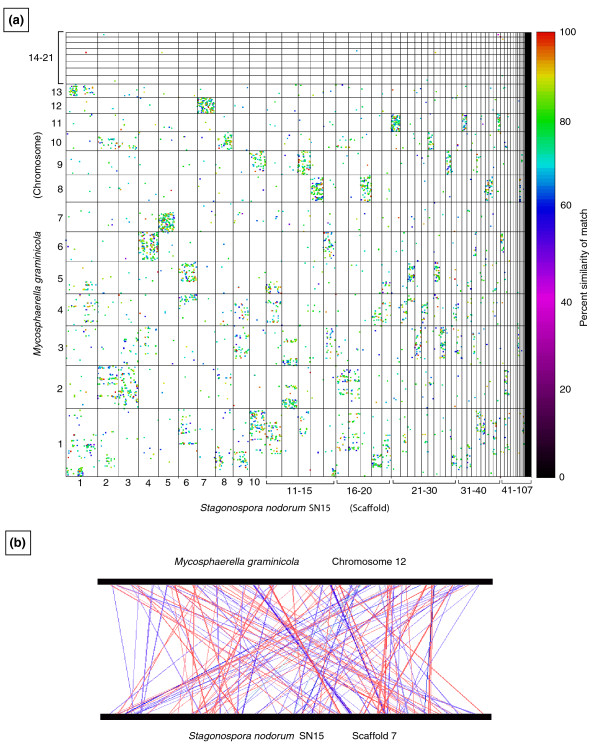
**Whole-genome dot-plot comparison between the Dothideomycetes species *Mycosphaerella graminicola *and *Phaeosphaeria nodorum***. **(a) **The six-frame translations of both genomes were compared via MUMmer 3.0. Homologous regions are plotted as dots, which are color coded for percent similarity as per the color bar (right). Chromosomes 1 to 21 from *M. graminicola *are displayed by decreasing size along the y axis and scaffolds 1 to 107 from *P. nodorum *are displayed along the x axis. Dots represent matching regions between translated scaffold sequences. Mesosyntenic regions appear as dots within boxes without any obvious diagonal lines. **(b) **Homology relationships between chromosome 12 of *M. graminicola *and scaffold 17 of *P. nodorum*. Red lines link parallel homologous pairs and blue lines link anti-parallel pairs.

The genome sequences of other Dothideomycetes species have become available recently, allowing whole-genome comparisons with relatively closely related taxa. We used the software tool MUMmer [37] to generate dot plots that compare the scaffolds of the *P. nodorum *assembly with the 21 finished chromosomes of *Mycosphaerella graminicola *[38,39] (Figure 2a). These species are classified respectively in the Pleosporales and the Capnodiales, order-level taxa within the Dothideomycetes (Figure 2), with an estimated divergence time of (very approximately) 300 million years ago (Mya). The figure is arranged with the chromosomes or scaffolds of each species arranged in size order along the axes. Dots correspond to regions of sequence similarity and are color-coded to indicate their degree of identity.

Our expectation was that we would see either dispersed diagonal lines or a completely random distribution of very short matches ('dots'). Instead, the dot plot shows a highly non-random distribution whereby dots from individual chromosomes of *M. graminicola *appear to be strongly associated with one or a few scaffolds of *P. nodorum*, indicated by 'boxes' within columns and rows. For example, dots corresponding to *P. nodorum *scaffold 7 were almost exclusively found within the box corresponding to *M. graminicola *chromosome 12. Reciprocally, dots corresponding to *M. graminicola *chromosome 12 appeared predominantly within the box corresponding to *P. nodorum *scaffold 7. The dots within this box did not fall on any obvious diagonal lines and were instead arranged quasi-randomly. When these two sequences were aligned (Figure 2b), lines joining regions of significant similarity were distributed quasi-randomly. The orientation of the genes (color coded as red for parallel and blue for inverted) also appeared to be random. The dot plots used six-frame back translations of the genomes. Similar results were obtained with raw nucleotide sequences or when validated genes were used (Additional file 2). This indicated that the majority of the dots corresponded to genes.

We call this pattern of dots-within-boxes 'mesosynteny'. The non-random distribution implies conservation of the gene content of scaffolds (and by implication, chromosomes) during evolution; hence, this is a form of synteny [40] that does not involve the retention of co-linearity as found in both macro- and microsynteny.

### Taxonomic distribution of mesosynteny across the fungal kingdom

**Figure 3:**
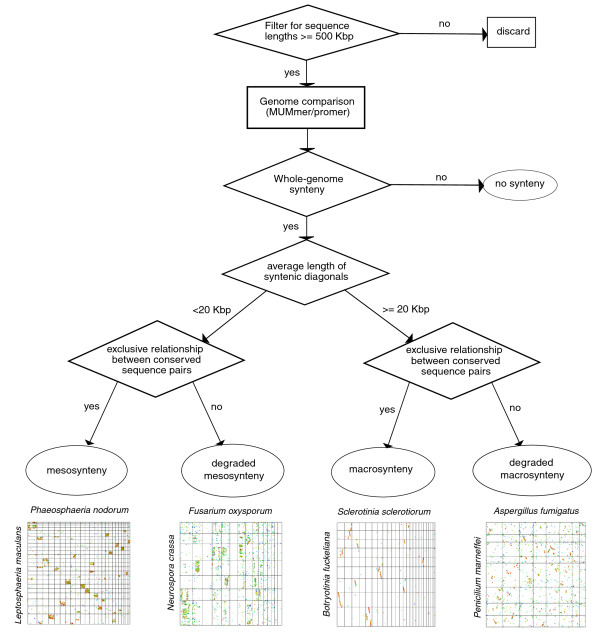
**The whole-genome synteny classification process**. Genome sequences of fewer than 500 kb were discarded before analysis. The results of MUMmer comparisons between a pair of genomes were tested for synteny (see Materials and methods). If a genome pair was determined to be syntenic at a whole-genome level, synteny was classified as 'mesosyntenic' or 'macrosyntenic' based on the average length of co-linear diagonals.

To test the extent and generality of mesosynteny within the fungi, the analysis was extended to other species within the Dothideomycetes, other classes within the Pezizomycotina and other fungal phyla. These comparisons were tested for chromosomal-scale genome conservation and were classified as macrosyntenic, mesosyntenic, or non-syntenic (Figure 3; Additional file 3).

Visual inspection of dot plots distinguished the comparisons neatly into three classes: no synteny, macrosynteny or mesosynteny. Quantifying the degree of synteny between species required the development of new statistical tests. Significant sequence conservation was tested between pairs of scaffolds by a one-tailed cumulative binomial test, requiring a probability of ≥0.99. The whole-genome conservation was defined as significant when ≥25% of the expected number of scaffold pairs (assuming perfect whole-genome synteny) were conserved. Species pairs showing synteny were classified as macro- or mesosynteny based on the average length of co-linear runs of sequence matches between both genomes; an average co-linear diagonal length of ≥20 kb was considered macrosyteny and < 20 kb was classified as mesosynteny (Additional file 3). Mesosynteny and macrosynteny were further categorized into 'degraded' or 'non-degraded' (Figure 3). Synteny was classified as degraded when significant clusters of 'dots' or 'lines' were found outside of the primary box (that is, for any given 'box', < 75% of the total length of conserved sequences within its corresponding rows and columns resided within the dominant box). Scaffolds shorter than 500 kb were excluded from these analyses.

**Figure 4:**
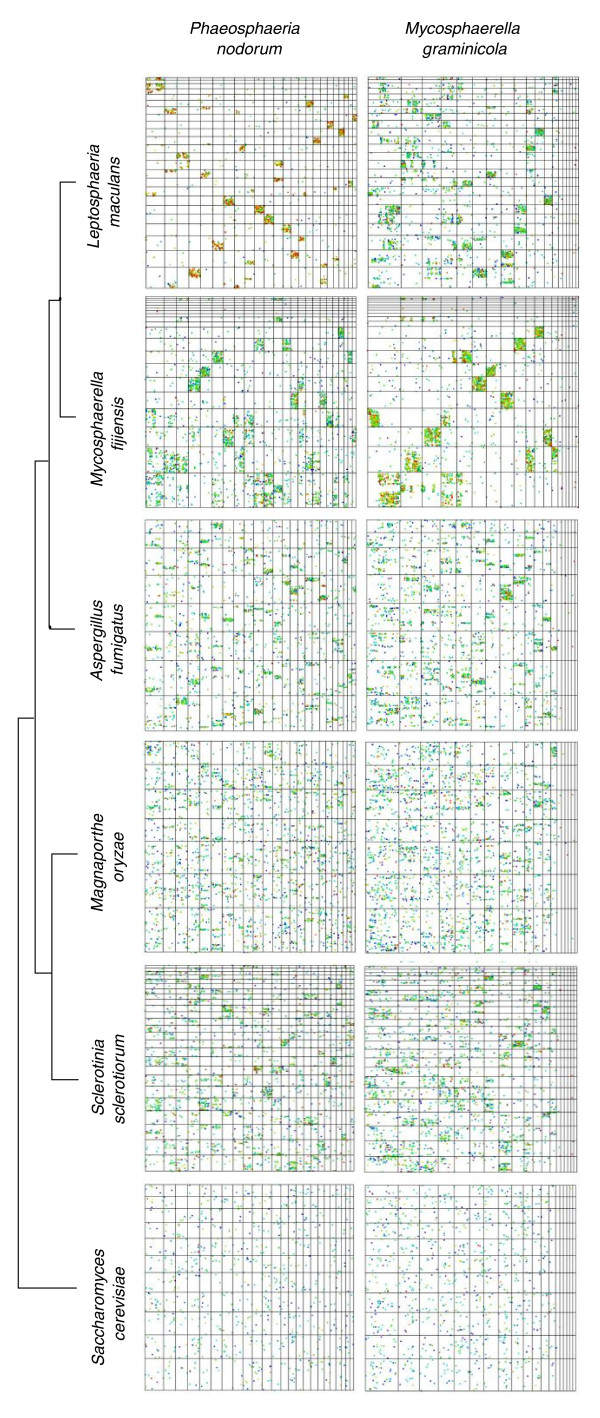
**Dot-plot comparisons between the class Dothideomycetes and related classes**. Scaffolds greater than 500 kb in length are ordered in ascending alpha-numeric order upwards along the y-axis and left-to-right along the x-axis. The orders Pleosporales (represented by *P. nodorum *and *L. maculans*) and Capnodiales (*Mycosphaerella *spp.) of the Dothideomycetes exhibit a tightly clustered pattern of mesosynteny between and within each order. This degrades into a mesosynteny-like pattern in comparisons between Dothideomycetes and the classes Eurotiomycetes (*A. fumigatus*), Sordariomycetes (*M. oryzae*) and Leotiomycetes (*S. sclerotiorum*). Clustered blocks can still be observed in these comparisons. The dot-plots comparing Dothideomycetes and Saccharomycetes (*S. cerevisiae*) appear to be random - that is, there was no synteny.

Dot-plot comparisons between the Dothideomycetes species *P. nodorum*, *M. graminicola*, *Mycosphaerella fijiensis *and *Leptosphaeria maculans *showed significant mesosynteny (Figure 4). The comparison between *P. nodorum *and *L. maculans *(both in the order Pleosporales) was especially striking (Additional file 2). The dot plot was dominated by matches of 80 to 100% similarity, compared to 60 to 80% in the case of *P. nodorum *versus the species in the Capnodiales, *M. graminicola *or *M. fijiensis*. The dots in the comparison between *P. nodorum *and *L. maculans *were almost exclusively restricted to single boxes within both rows and columns. As before, there was no indication of the diagonal lines characteristic of macrosynteny. This pattern of nearly exclusive dots within single boxes was also observed when comparisons were made between these genomes and the other released but so far unpublished Dothideomycetes genomes available via the JGI and Broad Institute web sites ([39,41,42] and data not shown).

Dothideomycetes species also showed a discernable level of mesosynteny-like conservation with species representing the classes Eurotiomycetes (*Aspergillus fumigatus*), and the Leotiomycetes (*Sclerotinia sclerotiorum*; *S. sclerotiorum *sequencing project [43]), but not with the Sordariomycetes (*Magnaporthe oryzae*) or the Saccharomycetes (*Saccharomyces cerevisiae*) (Figure 4; Additional file 1). Comparisons of *P. nodorum *and *M. graminicola *with *A. fumigatus *and *S. sclerotiorum *had a statistically significant non-random distribution of dots within boxes. In contrast to intra-Dothideomycetes comparisons, dots appeared in multiple boxes within a row and column. This is an example of degraded mesosynteny. Comparisons between Dothideomycetes and *M. oryzae *(Sordariomycetes) and the yeast *S. cerevisiae *(Saccharomycetes) failed to find a statistically significant degree of synteny, reflected in the apparently random distribution of dots. These comparisons had an average of 1 and 0 sequences with binomial probabilities of significant sequence conservation >0.99. No statistically significant syntenic relationships were found when either *M. oryzae *or any yeast was compared with other filamentous fungal genomes.

**Figure 5:**
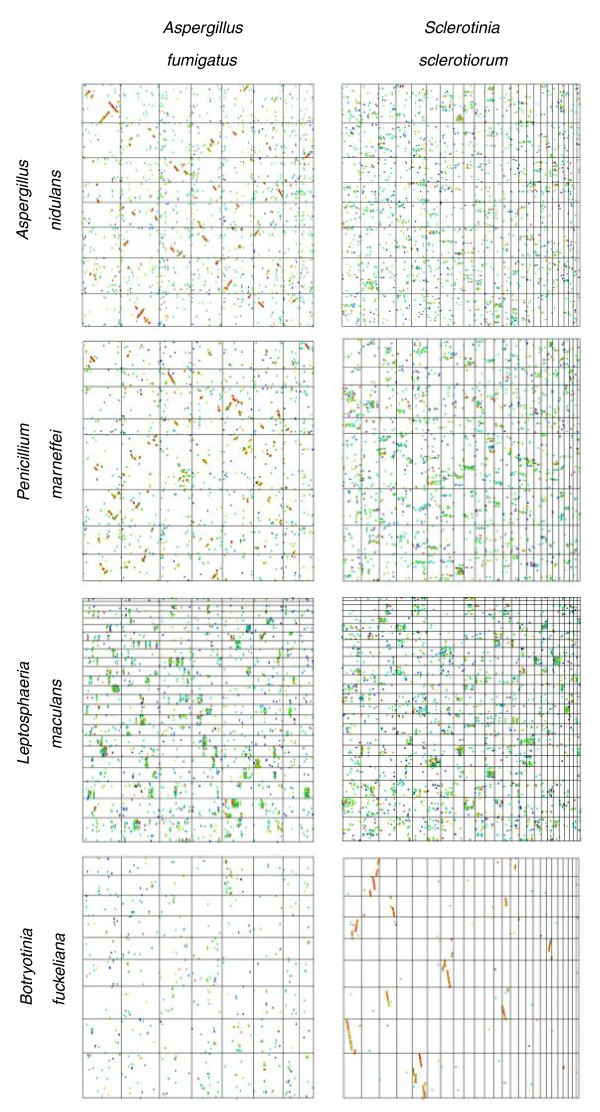
**Dot-plot comparisons between representatives of the classes Eurotiomycetes and Leotiomycetes and related classes**. The orders Eurotiales (*Aspergillus *spp.) and Onygenales (*P. marneffei*) of the Eurotiomycetes exhibit degraded macrosynteny between and within each order. The Leotiomycetes exhibit macrosynteny between species of the order Helotiales. A mesosynteny-like pattern is observed in comparisons between the Eurotiomycetes, Leotiomycetes and the more distantly related class Dothideomycetes (*L. maculans*).

A similar series of dot-plot comparisons between the class Eurotiomycetes and Leotiomycetes and species from classes of the Ascomycota is shown in Figure 5. The test species are *A. fumigatus *and *S. sclerotiorum*. The comparisons between *S. sclerotiorum *and *Botryotinia fuckeliana *exhibited a highly conserved pattern with many obvious diagonal lines made up of red and yellow dots representing highly similar (90 to 100%) sequence pairs. The average length of co-linear regions was much greater than 20 kb. This is a classical macrosyntenic pattern reflecting very recent divergence between these closely related genera. A weaker macrosyntenic pattern was observed between *A. fumigatus *and *Penicillium marneffei*, two species in the Eurotiales. Less than 25% of matches in columns and rows resided within a single box, characteristic of degraded macrosynteny. Comparisons between *A. fumigatus *and *S. sclerotiorum *and the Dothideomycetes, represented by *L. maculans*, revealed degraded mesosynteny. This was also observed between *S. sclerotiorum *and the two members of the Eurotiales, *A. fumigatus *and *P. marneffei*.

**Figure 6:**
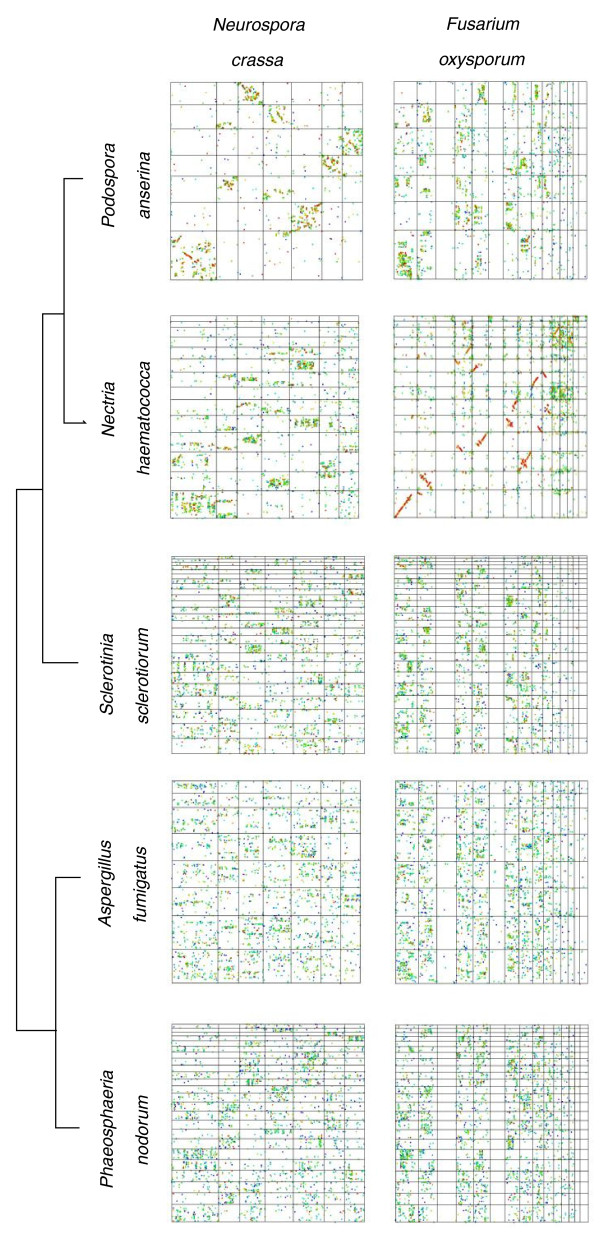
**Dot-plot comparisons between two members of the class Sordariomycetes and related classes**. The orders Sordariales (*N*. crassa and *P. anserina*) and Hypocreales (*F. oxysporum *and *N. haematococca*) of the Sordariomycetes generally exhibit mesosynteny-like conservation. The closely related pair of Hypocreales species *Fusarium oxysporum *and *Nectria haematococca *exhibit mostly macrosynteny with mesosynteny between a few chromosomes. Comparisons between the Sordariales and Hypocreales with the classes Leotiomycetes, Eurotiomycetes and Dothideomycetes exhibit degraded mesosynteny. Lack of synteny between the conditionally dispensable chromosomes 3, 6, 14 and 15 of *F. oxysporum *(excluding *N. haematococca*) with chromosomes of any other species is evident by an absence of dots in those comparisons.

The Sordariomycetes *Fusarium oxysporum *exhibited mixed patterns of synteny in comparisons between species from the related orders Sordariales and Hypocreales and from other classes in the Pezizomycotina (Figure 6; Additional file 4). Striking macrosynteny was observed between chromosomes 1, 2, 4, 5 and 7 to 10 of *F. oxysporum *and chromosomes 1 to 6 and 7 to 10 of *Nectria haematococca*. Parts of chromosomes 3, 6 and 11 to 14 of *F. oxysporum *exhibited a mesosyntenic pattern with chromosomes 7 and 11 to 14 of *N. haematococca*. Mesosynteny was strongest between *N. haematococca *chromosome 14 and parts of *F. oxysporum *chromosomes 3, 6, 14 and 15. Degraded mesosynteny was observed between *F. oxysporum *and *Neurospora crassa*, *S. sclerotiorum*, *A. fumigatus *and with *P. nodorum*. However, in all comparisons (excluding *N. haematococca*), dots were conspicuously absent from rows corresponding to *F. oxysporum *chromosomes 3, 6, 14 and 15 (Figure 6).

The comparison between *N. crassa *and *Podospora anserina *(order Sordariales) showed a dominant pattern of mesosynteny, with some macrosyntenic regions particularly between the largest chromosome of both species [20]. *N. crassa *exhibited degraded mesosynteny when compared to *S. sclerotiorum*, *A. fumigatus *and *P. nodorum *(Figure 6).

We expanded these comparisons beyond the classes presented above to include additional species of the sub-phyla Saccharomycotina and Taphrinomycotina within the phylum Ascomycota and species from the phyla Basidiomycota, Blastocladiomycota and Chytridiomycota (Table 1; Figure 1). Non-filamentous Ascomycetes (classes Saccharomycetes and Schizosaccharomycetes) exhibited either macrosynteny or no synteny within their respective classes and no synteny with other fungal classes (Additional file 5). The class Agaricomycetes exhibited degraded macrosynteny between species within the class and no synteny with other fungal classes (Additional file 6). No non-Pezizomycotina taxa showed any level of synteny when compared to species of a different class (Table 1; Additional file 1).

## Discussion

A novel and unexpected mode of chromosome-level sequence conservation, which we have called mesosynteny, has been detected between species of filamentous Ascomycetes, and in particular the Dothideomycetes. Mesosynteny implies the conservation of gene content within chromosomes but without conservation of gene order or orientation. It contrasts markedly with the macrosynteny observed commonly in plants and animals and the absence of synteny seen in other eukaryotes such as distantly related yeast species. The cause of mesosyntenic chromosomal evolution is not known. However, a mesosyntenic pattern would be expected to occur if intra-chromosomal recombination (including inversions) occurred significantly more frequently than inter-chromosomal recombinational events such as translocations.

Mesosynteny is distinct from macrosynteny. Macrosynteny would be expected to arise when the predominant modes of chromosomal evolution are inter-chromosomal recombination and gene loss. These considerations suggest that different patterns of mutagenic events can lead either to mesosynteny or to macrosynteny as chromosomes evolve following a speciation event.

Mesosynteny also is distinct from microsynteny, which is characterized by co-linearity between clusters of two to about ten genes with both order and orientation conserved. Earlier comparisons of synteny in related filamentous fungi frequently found clusters of genes with related functions but without retention of gene order or orientation. An example is the quinate cluster, which is conserved across species of the Ascomycetes [21,44]. This pattern of shuffled cluster retention is akin to what we observe at the whole-chromosome level.

Our results suggest that mesosyntenic chromosomal conservation is restricted to the Pezizomycotina and is most pronounced in the Dothideomycetes [45]. The Dothideomycetes are the only group to exhibit non-degraded mesosynteny between species of the different genera (estimated to have diverged approximately 120 to 150 Mya) and orders (approximately 300 Mya). A recognizable yet degraded form of mesosynteny was found between many species of Pezizomycotina outside the Dothideomycetes. The estimated time of divergence within Dothideomycetes orders are comparable to other orders within the Pezizomycotina that exhibited either degraded mesosynteny or no detectable synteny (Table 1) [38]. No mesosynteny was observed in any of the fungal groups outside of the Pezizomycotina that were surveyed: yeasts, Basidiomycetes, Blastocladiomycetes and Chytridiomycetes. The evolutionary separation between these groups and the Dothideomycetes (500 to 650 Mya) [38] may be so great that both mesosynteny and macrosynteny have decayed below the limit of detection. To our knowledge, mesosynteny has not been observed in non-fungal eukaryotes. Superficially similar dot-plots have been occasionally observed in comparisons of chordate genomes [45] but appear to be due to the amplification of paralogous copies of genes within chromosomes. Overall, either macrosynteny or no synteny has been found outside the Pezizomycotina.

Chromosomal conservation akin to mesosynteny had been observed previously in a number of inter-species comparisons within the Pezizomycotina, but its full extent was not analyzed. These include comparisons between the Pezizomycetes *Tuber melanosporum *and the Eurotiomycetes *Coccidioides immitus *[46] and the Sordariomycetes *P. anserina *and *N. crassa *[20]. As *N. crassa *and *P. anserina *are heterothallic, the authors suggested that the observed conservation may be specific to out-crossing (heterothallic) fungi. However, evidence from this study suggests otherwise as mesosynteny was observed between both heterothallic and homothallic species (Table 1; Additional files 3 and 5). For example, two homothallic Sordariomycetes species, which diverged approximately 225 Mya [38], exhibited degraded mesosynteny (*Fusarium graminearum *and *Chaetomium globosum*; Additional file 7).

Mesosynteny was observed in species both with and without (*F. oxysporum *[47] and *Penicillium marneffei *[48]) a known sexual stage. It may be that sexual crossing has been lost relatively recently in these species. Nonetheless, this finding suggests that mesosyntenic relationships were not quickly lost in the absence of meiosis. Amongst the mesosyntenic Pezizomycotina, mesosynteny was weakest in comparisons against the *M. oryzae *genome (Figures 5 and 6; Table 1). *M. oryzae *is believed to exist in nature in purely asexual lineages. The sequenced isolate of *M. oryzae *was a fertile derivative of two asexual lineages [19]. We speculate that a history of asexual reproduction and/or the process of laboratory domestication may have destroyed the remnants of mesosynteny in this isolate. This hypothesis could be tested by comparisons with genome sequences from additional isolates of *M. oryzae *or related species in the Magnaporthales.

In some species there was an uneven distribution of syntenic relationships between different chromosomes. The genome of *M. graminicola *has been finished [39] and comprises 21 chromosomes, the eight smallest of which have been shown to be dispensable [49]. These dispensable chromosomes displayed little sequence conservation with genes from any other species, and therefore no detectable synteny of any type (Figure 4). In contrast, the *M. graminicola *core chromosomes exhibited a typical mesosyntenic pattern in most comparisons with other Pezizomycotina species. Similarly, the conditionally dispensable chromosomes (CDCs) of *F. oxysporum *(3, 6, 14 and 15) showed no synteny with almost all species tested. All of these supernumerary chromosomes are thought to have originated by lateral transfer from unknown donor species [39,50]. Whether the lack of synteny of most supernumerary chromosomes is because they come from distantly related species or because they evolve more rapidly than core chromosomes is not known.

Surprisingly, CDC 14 of *N. haematococca *was mesosyntenic to *F. oxysporum *CDCs (chromosome 14 and the terminal end of chromosomes 3 and 6; Figure 6; Additional file 4). This is in contrast to the core chromosomes of each species, which exhibited macrosynteny and to previous comparisons that had indicated that these CDCs were non-syntenic. A comparison of the *F. oxysporum *genome with the closely related *Fusarium verticillioides *indicated that the *F. oxysporum *CDCs were not syntenic [50]. A possible explanation for this phenomenon is that mutations and rearrangements in supernumerary chromosomes accumulate more rapidly because these chromosomes are rarely required for survival. Faster accumulation of mutations potentially coupled with origins in distantly related donor species may allow the sequences of supernumerary chromosomes to diverge to the point where no sequence similarity remains (as in *M. graminicola*). The occurrence of mesosyntenic rearrangement in *F. oxysporum *and *N. haematococca *may also be related to the origin of their CDCs. These may have arisen in their common ancestor from a single chromosome, which subsequently mutated and broke into smaller chromosomes. Alternatively, they may have been recently transferred laterally from a common (or closely related) donor.

Whole-genome shotgun sequencing involves the generation of many short DNA reads that are assembled into longer segments. Macrosyntenic relationships are commonly used to assist the assembly and finishing of fragmented genome sequences, particularly in prokaryotic genomes. Sequences that are macrosyntenic to a long sequence of a closely related genome can be confidently hypothesized to be joined physically. Mesosynteny between a new genome assembly with a reference genome also may be used to suggest which scaffolds are juxtaposed. This could significantly reduce the cost and complexity of assembling and finishing genomes. To test whether mesosynteny could be used to predict scaffold joins in genomic sequences, early and late assemblies of the *M. graminicola *genome were analyzed to determine whether the joining of contigs or scaffolds in the finished genome could have been predicted by mesosyntenic relationships of the draft genome to *P. nodorum *[39]. Mesosynteny was remarkably successful in predicting separate scaffolds that should be joined and for identifying mis-joins in the initial assembly. This approach has the potential to assist with assembly and finishing of other genomes within the Pezizomycotina.

## Conclusions

We have unearthed a novel mode of evolution in which chromosomes retain their content but shuffle the order and orientation of genes. We propose to call this phenomenon mesosynteny. What is the origin and mechanism of mesosynteny? The phenomenon is observed only in the Pezizomycotina and especially in the Dothideomycetes. The Dothideomycetes sequenced to date have several (ten or more) relatively small chromosomes, hinting at the ubiquity of supernumerary chromosomes within this taxon. The Pezizomycotina exhibit repeat-induced point mutation and higher frequencies of lateral gene transfer compared to other fungi [15,51]. Are these phenomena causally related?

The mechanism for mesosynteny may occur through a high frequency of inversions during meiosis. Whether the Dothideomycetes have a higher propensity for inversions is not known but should be the subject of further investigation. Alternatively, lateral gene transfer may be the driving force behind mesosynteny. The mechanism of lateral gene transfer is not well understood, but recent evidence suggests that the sequence transferred can be very large, even up to the size of entire chromosomes [49,52]. Fungi are capable of fusing with other fungal species through either conidial or hyphal anastomosis tubes [53]. Fusion can lead to exchange of nuclei and the transient formation of heterkaryotic strains. If the transferred DNA carried a gene that was beneficial to the recipient species, the chromosome (or a large section) carrying this gene may be retained whilst other donated chromosomes would be lost. As mesosynteny tends to retain genes on the same chromosomes, a recipient species may be able to accept a substitute chromosome from a reasonably closely related species without major disruption of phenotype. Recombination between the new and old chromosome would shuffle the order and orientation of genes, with remnant duplicated genes being removed in further cycles of repeat-induced point mutation. Recombinants with a complete core gene content plus any advantageous laterally transferred genes would then be selected, resulting in the mesosyntenic pattern of chromosomal conservation we see today. Mesosynteny may, therefore, be an adaptive mechanism that both allowed and resulted from lateral acquisition of large chromosomal sections.

## Materials and methods

### Whole-genome comparisons

The synteny classification method is outlined in Figure 3. Genome sequence assemblies of the species listed in Figure 1 were obtained from the sources described in Additional file 1. Phylogenetic data (Figure 1; Table 1) were inferred from previous publications [1,28,54,55]. Individual sequences (contigs, scaffolds or chromosomes) less than 500 kbp in length were discarded from the analysis. Whole-genome comparisons were performed using promer (MUMmer 3.0, [37]) with the '--mum' parameter. Promer outputs were filtered for repetitive matches using the program 'delta-filter' (MUMmer 3.0) with the '-g' parameter. Genome dot plots were generated using 'mummerplot' (MUMmer 3.0) and coordinates of promer matches were derived from filtered promer outputs using the 'show-coords' program (MUMmer 3.0).

### Determination of significant sequence conservation

For the purposes of this study, only synteny observable at a whole-genome level was considered. For a given pair of genomes (genome A and genome B), all combinations of their sequence (contigs, scaffolds or chromosomes) pairs (one sequence from genome A (sequence A) and one from genome B (sequence B)) were tested for significant conservation. Lengths of conserved regions in sequences A and B were derived from MUMmer outputs. The probability of synteny (P_syn_) for sequence pairs was calculated via a one-tailed cumulative binomial test:

where x = (Length conserved in sequence A × Length conserved in sequence B)/(Length of sequence A × Length of sequence B); *n *= 100; p = (Total length conserved in Genome A × Total length conserved in Genome B)/(Total length genome A × Total length genome B). P_syn _was required to be ≥ 0.99 to indicate significant amounts of sequence conservation between a sequence pair.

### Analysis of syntenic regions between conserved sequences

The lengths of syntenic regions were analyzed for significantly conserved sequence pairs. Extended co-linearity of sequence matches visible as uninterrupted diagonal lines on a dot plot was used as an indicator of macrosynteny (Additional file 1). Dot plots between sequence pairs were considered as individual scatter plots. Promer matches between a pair of sequences were converted into a series of points on the scatter plot, with a point added every 1 kb along each match. R^2 ^values were calculated along the axis of sequence A in 20-kb windows (incrementing along by 2 kb). A window was considered to be co-linear if it contained a minimum of 15 data points with an R^2 ^≥ 0.9 The end coordinates of co-linear windows were subsequently modified to exclude the coordinate range of overlapping non-co-linear windows. The data points of co-linear windows within 50 kb of one another were combined (including intermediate data points if not overlapping) and were merged into larger co-linear windows if (Slope of window 1/Slope of window 2) > 0.8 and < 1.2. The start and end points of co-linear windows with a length of ≥ 5 kb were used as the coordinates of 'syntenic regions'. The same process was repeated along the axis of sequence B.

### Classification of synteny type

Whole-genome synteny was identified by the 'significant pair ratio' statistic, which is an indicator of the proportion of conserved sequences relative to the expected number of conserved sequences. The significant pair ratio was determined by:

where N_scp _is the number of significantly conserved pairs between genomes A and B; S_a _is the number of sequences in genome A ≥ 500 kb; and S_b _is the number of sequences in genome B ≥ 500 kb.

Whole-genome synteny was identified when the significant pair ratio was ≥ 0.25. Genome pairs failing this criterion were classified as 'non-syntenic'. Genome pairs passing the test for whole-genome synteny were sub-categorized as either macrosyntenic or mesosyntenic, defined by an average length of syntenic regions (combined between both compared genomes) of greater than or less than 20 kb, respectively. Synteny type was further categorized into 'degraded' or 'non-degraded' based on the statistic 'pair exclusivity'. For a given sequence pair, consisting of sequence A of genome A and sequence B of genome B, the 'pair exclusivity' was calculated by:

where C_ab _is the total length of conserved matches between sequences A and B; C_Ab _is the total length of conserved matches for sequence A and all sequences of genome B; and C_aB _is the total length of conserved matches for sequence B and all sequences of genome A.

Synteny was classified as 'degraded' if the maximum value of all pair exclusivities was less than 0.75.

## Abbreviations

CDC: conditionally dispensable chromosome; Mya: million years ago.

## Authors' contributions

RPO and JKH conceived and designed the study. JKH developed algorithms and performed mesosynteny analyses. JKH and RPO wrote the manuscript. TR, BJH, GHJK and SBG contributed data. JKH, RPO, TR, BJH, GHJK and SBG edited the manuscript. All authors read and approved the final manuscript.

## Supplementary Material

Additional file 1 **Supplementary Figure S1**. The origins of the different types of syntenic relationships. Immediately after a speciation event, equivalent chromosomes in two daughter species retain the gene content, order and orientation of the parent species. **(a) **Diagrammatic representation of a chromosome with sequential elements A to Z. **(b) **A dot plot comparing the chromosomes in (a), with letters substituted for dots. The unbroken series of letters on the diagonal indicates macrosynteny. **(c, d) **Loss of sequences from each chromosome (c) will degrade the diagonal co-linearity when visualized as a dot plot (d). Click here for file

Additional file 2 **Supplementary Figure S2**. **(a, b) **Correspondence between promer-derived dot plots (a) and blastp-derived protein comparisons of annotated genes (b) between *Phaeosphaeria nodorum *and *Leptosphaeria maculans*. Sequence pairs ('boxes') in (a) containing non-random distributions of 'dots' correspond to those in (b), indicating that the back-translated genome matches in (a) correspond to regions of conserved gene content. Click here for file

Additional file 3 **Supplementary File 1**. Predictions of synteny between all species involved in this study in an Excel file. Click here for file

Additional file 4 **Supplementary Figure S3**. Presence of both macrosyntenic and mesosyntenic conservation patterns between the genomes of *Fusarium oxysporum *and *Nectria haematococca*. Core chromosomes (indicated by black bars along the axes) are macrosyntenic between the two species. Dispensable chromosomes (red bars along the axes) are either non-syntenic (*N. haematococca *chromosomes 15 to 17) or mesosyntenic (*N. haematococca *chromosomes 7 and 11 to 14, *F. oxysporum *chromosome 14). The majority of chromosomes 3 and 6 of *F. oxysporum *had no similarity to the chromosomes of *N. haematococca *except for regions near their telomeres. Click here for file

Additional file 5 **Supplementary Figure S4**. Degradation of whole-genome synteny in the classes Saccharomycetes and Schizosaccharomyces. Whole-genome dot plots have been limited to scaffolds or chromosomes greater than 500 kb. Species of the *Saccharomyces *and *Schizosaccharomyces *do not exhibit whole-genome conservation with each other. Certain species within each class exhibit macrosynteny whereas others exhibit no synteny. Click here for file

Additional file 6 **Supplementary Figure S5**. Degradation of whole-genome synteny between a member of the class Agaricales and related orders. Whole-genome dot plots have been limited to scaffolds or chromosomes greater than 500 kb. Species in the Agaricales exhibited macrosynteny with each other. However, the Agaricales exhibited no synteny with the closest related classes represented in this study, the Exobasidiomycetes and Pucciniomycetes. Click here for file

Additional file 7 **Supplementary Figure S6**. Evidence of degraded mesosynteny between the genomes of two homothallic Sordariomycete species, *Fusarium graminearum *(order Hypocreales) and *Chaetomium globosum *(order Sordariales). These two species are estimated to have diverged approximately 225 Mya. Sequence matches (dots) are arranged in blocked clusters typical of mesosynteny. Chromosomes and scaffolds do not share a one-to-one relationship, with multiple mesosyntenic clusters appearing in the same row or column. Click here for file

## References

[B1] Schoch CL Sung GH Lopez-Giraldez F Townsend JP Miadlikowska J Hofstetter V Robbertse B Matheny PB Kauff F Wang Z Gueidan C Andrie RM Trippe K Ciufetti LM Wynns A Fraker E Hodkinson BP Bonito G Groenewald JZ Arzanlou M de Hoog GS Crous PW Hewitt D Pfister DH Peterson K Gryzenhout M Wingfield MJ Aptroot A Suh SO Blackwell M The Ascomycota tree of life: a phylum-wide phylogeny clarifies the origin and evolution of fundamental reproductive and ecological traits. Syst Biol 2009 58 224 239 10.1093/sysbio/syp020 20525580

[B2] Sims GE Jun SR Wu GA Kim SH Alignment-free genome comparison with feature frequency profiles (FFP) and optimal resolutions. Proc Natl Acad Sci USA 2009 106 2677 2682 10.1073/pnas.0813249106 19188606 PMC2634796

[B3] McLysaght A Enright AJ Skrabanek L Wolfe KH Estimation of synteny conservation and genome compaction between pufferfish (*Fugu*) and human. Yeast 2000 17 22 36 10.1002/(SICI)1097-0061(200004)17:1<22::AID-YEA5>3.0.CO;2-S 10797599 PMC2447035

[B4] Pennacchio LA Insights from human/mouse genome comparisons. Mamm Genome 2003 14 429 436 10.1007/s00335-002-4001-1 12925891

[B5] Kohn M Kehrer-Sawatzki H Vogel W Graves JA Hameister H Wide genome comparisons reveal the origins of the human X chromosome. Trends Genet 2004 20 598 603 10.1016/j.tig.2004.09.008 15522454

[B6] Cannon SB Sterck L Rombauts S Sato S Cheung F Gouzy J Wang X Mudge J Vasdewani J Schiex T Spannagl M Monaghan E Nicholson C Humphray SJ Schoof H Mayer KF Rogers J Quetier F Oldroyd GE Debelle F Cook DR Retzel EF Roe BA Town CD Tabata S Van de Peer Y Young ND Legume genome evolution viewed through the *Medicago truncatula *and *Lotus japonicus *genomes. Proc Natl Acad Sci USA 2006 103 14959 14964 10.1073/pnas.0603228103 17003129 PMC1578499

[B7] Phan HT Ellwood SR Hane JK Ford R Materne M Oliver RP Extensive macrosynteny between Medicago truncatula and Lens culinaris ssp. culinaris. Theor Appl Genet 2007 114 549 558 10.1007/s00122-006-0455-3 17119911

[B8] Shultz JL Ray JD Lightfoot DA A sequence based synteny map between soybean and *Arabidopsis thaliana*. BMC Genomics 2007 8 8 10.1186/1471-2164-8-8 17210083 PMC1780048

[B9] Parkin AP Lydiate DJ Trick M Assessing the level of collinearity between *Arabidopsis thaliana *and *Brassica napus *for *A. thaliana *chromosome 5. Genome 2002 45 356 366 10.1139/g01-160 11962633

[B10] Ma B Synteny between *Brachypodium distachyon *and *Hordeum vulgare *as revealed by FISH. Chromosome Res 2010 18 841 850 10.1007/s10577-010-9166-3 21104310

[B11] Zhang Y Schoch CL Fournier J Crous PW de Gruyter J Woudenberg JH Hirayama K Tanaka K Pointing SB Spatafora JW Hyde KD Multi-locus phylogeny of Pleosporales: a taxonomic, ecological and evolutionary re-evaluation. Stud Mycol 2009 64 85 102S5 10.3114/sim.2009.64.04 20169024 PMC2816967

[B12] Marande W López-García P Moreira D Eukaryotic diversity and phylogeny using small- and large-subunit ribosomal RNA genes from environmental samples. Environ Microbiol 2009 11 3179 3188 10.1111/j.1462-2920.2009.02023.x 19678831

[B13] Wang H Guo S Huang M Thorsten LH Wei J Ascomycota has a faster evolutionary rate and higher species diversity than Basidiomycota. Sci China 2010 53 1163 1169 10.1007/s11430-010-4011-2 20953937

[B14] Oliver RP Solomon PS Recent fungal diseases of crop plants: is lateral gene transfer a common theme? Mol Plant Microbe Interact 2008 21 287 293 10.1094/MPMI-21-3-0287 18257678

[B15] Marcet-Houben M Gabaldon T Acquisition of prokaryotic genes by fungal genomes. Trends Genet 2010 26 5 8 10.1016/j.tig.2009.11.007 19969385

[B16] Friesen TL Stukenbrock EH Liu Z Meinhardt S Ling H Faris JD Rasmussen JB Solomon PS McDonald BA Oliver RP Emergence of a new disease as a result of interspecific virulence gene transfer. Nat Genet 2006 38 953 956 10.1038/ng1839 16832356

[B17] Chibana H Oka N Nakayama H Aoyama T Magee BB Magee PT Mikami Y Sequence finishing and gene mapping for *Candida albicans *chromosome 7 and syntenic analysis against the *Saccharomyces cerevisiae *genome. Genetics 2005 170 1525 1537 10.1534/genetics.104.034652 15937140 PMC1449773

[B18] Borkovich KA Alex LA Yarden O Freitag M Turner GE Read ND Seiler S Bell-Pedersen D Paietta J Plesofsky N Plamann M Goodrich-Tanrikulu M Schulte U Mannhaupt G Nargang FE Radford A Selitrennikoff C Galagan JE Dunlap JC Loros JJ Catcheside D Inoue H Aramayo R Polymenis M Selker EU Sachs MS Marzluf GA Paulsen I Davis R Ebbole DJ Lessons from the genome sequence of *Neurospora crassa*: Tracing the path from genomic blueprint to multicellular organism. Microbiol Mol Biol Rev 2004 68 1 108 10.1128/MMBR.68.1.1-108.2004 15007097 PMC362109

[B19] Dean RA Talbot NJ Ebbole DJ Farman ML Mitchell TK Orbach MJ Thon M Kulkarni R Xu JR Pan H Read ND Lee YH Carbone I Brown D Oh YY Donofrio N Jeong JS Soanes DM Djonovic S Kolomiets E Rehmeyer C Li W Harding M Kim S Lebrun MH Bohnert H Coughlan S Butler J Calvo S Ma LJ The genome sequence of the rice blast fungus *Magnaporthe grisea*. Nature 2005 434 980 986 10.1038/nature03449 15846337

[B20] Espagne E Lespinet O Malagnac F Da Silva C Jaillon O Porcel BM Couloux A Aury JM Segurens B Poulain J Anthouard V Grossetete S Khalili H Coppin E Dequard-Chablat M Picard M Contamine V Arnaise S Bourdais A Berteaux-Lecellier V Gautheret D de Vries RP Battaglia E Coutinho PM Danchin EG Henrissat B Khoury RE Sainsard-Chanet A Boivin A Pinan-Lucarre B The genome sequence of the model ascomycete fungus *Podospora anserina*. Genome Biol 2008 9 R77 10.1186/gb-2008-9-5-r77 18460219 PMC2441463

[B21] Hane JK Lowe RG Solomon PS Tan KC Schoch CL Spatafora JW Crous PW Kodira C Birren BW Galagan JE Torriani SF McDonald BA Oliver RP Dothideomycete plant interactions illuminated by genome sequencing and EST analysis of the wheat pathogen *Stagonospora nodorum*. Plant Cell 2007 19 3347 3368 10.1105/tpc.107.052829 18024570 PMC2174895

[B22] Nierman WC Pain A Anderson MJ Wortman JR Kim HS Arroyo J Berriman M Abe K Archer DB Bermejo C Bennett J Bowyer P Chen D Collins M Coulsen R Davies R Dyer PS Farman M Fedorova N Fedorova N Feldblyum TV Fischer R Fosker N Fraser A Garcia JL Garcia MJ Goble A Goldman GH Gomi K Griffith-Jones S Genomic sequence of the pathogenic and allergenic filamentous fungus *Aspergillus fumigatus*. Nature 2005 438 1151 1156 10.1038/nature04332 16372009

[B23] Machida M Terabayashi Y Sano M Yamane N Tamano K Payne GA Yu J Cleveland TE Nierman WC Genomics of industrial Aspergilli and comparison with toxigenic relatives. Food Addit Contam Part A Chem Anal Control Expo Risk Assess 2008 25 1147 1151 18798040 10.1080/02652030802273114

[B24] Machida M Asai K Sano M Tanaka T Kumagai T Terai G Kusumoto K Arima T Akita O Kashiwagi Y Abe K Gomi K Horiuchi H Kitamoto K Kobayashi T Takeuchi M Denning DW Galagan JE Nierman WC Yu J Archer DB Bennett JW Bhatnagar D Cleveland TE Fedorova ND Gotoh O Horikawa H Hosoyama A Ichinomiya M Igarashi R Genome sequencing and analysis of *Aspergillus oryzae*. Nature 2005 438 1157 1161 10.1038/nature04300 16372010

[B25] Pel HJ de Winde JH Archer DB Dyer PS Hofmann G Schaap PJ Turner G de Vries RP Albang R Albermann K Andersen MR Bendtsen JD Benen JA van den Berg M Breestraat S Caddick MX Contreras R Cornell M Coutinho PM Danchin EG Debets AJ Dekker P van Dijck PW van Dijk A Dijkhuizen L Driessen AJ d'Enfert C Geysens S Goosen C Groot GS Genome sequencing and analysis of the versatile cell factory *Aspergillus niger *CBS 513.88. Nat Biotechnol 2007 25 221 231 10.1038/nbt1282 17259976

[B26] Maizel JV Jr Lenk RP Enhanced graphic matrix analysis of nucleic acid and protein sequences. Proc Natl Acad Sci USA 1981 78 7665 7669 10.1073/pnas.78.12.7665 6801656 PMC349330

[B27] Solomon PS Lowe RGT Tan KC Waters ODC Oliver RP *Stagonospora nodorum*: cause of stagonospora nodorum blotch of wheat. Mol Plant Pathol 2006 7 147 156 10.1111/j.1364-3703.2006.00326.x 20507435

[B28] Schoch CL Crous PW Groenewald JZ Boehm EW Burgess TI de Gruyter J de Hoog GS Dixon LJ Grube M Gueidan C Harada Y Hatakeyama S Hirayama K Hosoya T Huhndorf SM Hyde KD Jones EB Kohlmeyer J Kruys A Li YM Lucking R Lumbsch HT Marvanova L Mbatchou JS McVay AH Miller AN Mugambi GK Muggia L Nelsen MP Nelson P A class-wide phylogenetic assessment of Dothideomycetes. Stud Mycol 2009 64 1 15S10 10.3114/sim.2009.64.01 20169021 PMC2816964

[B29] Cooley RN Caten CE Variation in electrophoretic karyotype between strains of *Septoria nodorum*. Mol Gen Genet 1991 228 17 23 1886607 10.1007/BF00282442

[B30] Bringans S Hane JK Casey T Tan KC Lipscombe R Solomon PS Oliver RP Deep proteogenomics; high throughput gene validation by multidimensional liquid chromatography and mass spectrometry of proteins from the fungal wheat pathogen *Stagonospora nodorum*. BMC Bioinformatics 2009 10 301 10.1186/1471-2105-10-301 19772613 PMC2753851

[B31] Casey T Solomon PS Bringans S Tan KC Oliver RP Lipscombe R Quantitative proteomic analysis of G-protein signalling in *Stagonospora nodorum *using isobaric tags for relative and absolute quantification. Proteomics 2010 10 38 47 10.1002/pmic.200900474 19882661

[B32] Tan K-C Heazlewood JL Millar AH Oliver RP Solomon PS Proteomic identification of extracellular proteins regulated by the *Gna1 *Gα subunit in *Stagonospora nodorum*. Mycol Res 2009 113 523 531 10.1016/j.mycres.2009.01.004 19284980

[B33] Oliver RP Solomon PS New developments in pathogenicity and virulence of necrotrophs. Curr Opin Plant Biol 2010 13 415 419 10.1016/j.pbi.2010.05.003 20684067

[B34] Liu Z Faris JD Oliver RP Tan KC Solomon PS McDonald MC McDonald BA Nunez A Lu S Rasmussen JB Friesen TL SnTox3 acts in effector triggered susceptibility to induce disease on wheat carrying the Snn3 gene. PLoS Path 2009 5 e1000581 10.1371/journal.ppat.1000581 PMC2736379 19806176

[B35] Friesen TL Faris JD Solomon PS Oliver RP Host-specific toxins: Effectors of necrotrophic pathogenicity. Cell Microbiol 2008 10 1421 1428 10.1111/j.1462-5822.2008.01153.x 18384660

[B36] Friesen TL Zhang Z Solomon PS Oliver RP Faris JD Characterization of the interaction of a novel *Stagonospora nodorum *host-selective toxin with a wheat susceptibility gene. Plant Physiol 2008 146 682 693 18065563 10.1104/pp.107.108761 PMC2245837

[B37] Kurtz S Phillippy A Delcher AL Smoot M Shumway M Antonescu C Salzberg SL Versatile and open software for comparing large genomes. Genome Biol 2004 5 R12 10.1186/gb-2004-5-2-r12 14759262 PMC395750

[B38] Rouxel T Grandaubert J Hane J Hoede C van de Wouw A Couloux A Dominguez V Anthouard V Bally P Bourras S Cozijnsen A Ciuffetti L Dimaghani A Duret L Fudal I Goodwin S Gout L Glaser N Kema G Lapalu N Lawrence C May K Meyer M Ollivier B Poulain J Turgeon G Tyler BM Vincent D Weissenbach J Amselem J The compartmentalized genome of Leptosphaeria maculans: diversification of effectors within genomic regions affected by Repeat Induced Point mutations. Nat Commun 2011 2 art.202 10.1038/ncomms1189 PMC3105345 21326234

[B39] Goodwin SB Ben M'Barek S Dhillon B Wittenberg A Crane CF Van der Lee TAJ Grimwood J Aerts A Antoniw J Bailey A Bluhm B Bowler J Bristow J Brokstein P Canto-Canche B Churchill A Conde-Ferràez L Cools H Coutinho PM Csukai M Dehal P Donzelli B Foster AJ Hammond-Kosack K Hane J Henrissat B Kilian A Koopmann E Kourmpetis Y Kuo A Finished genome of *Mycosphaerella graminicola *reveals stealth pathogenesis and dispensome structure. PLoS Genetics -D-10-00112R2 2011 10.1371/journal.pgen.1002070 PMC3111534 21695235

[B40] Passarge E Horsthemke B Farber RA Incorrect use of the term synteny. Nat Genet 1999 23 387 10581019 10.1038/70486

[B41] Broad Institute http://www.broadinstitute.org/

[B42] DOE Joint Genome Institute http://www.jgi.doe.gov/

[B43] *S. sclerotiorum *Sequencing Project http://www.broadinstitute.org/annotation/genome/sclerotinia_sclerotiorum/Info.html

[B44] Giles NH Geever RF Asch DK Avalos J Case ME Organization and regulation of the qa (quinic acid) genes in *Neurospora crassa *and other fungi. J Hered 1991 82 1 7 1825499 10.1093/jhered/82.1.1

[B45] Putnam NH Butts T Ferrier DEK Furlong RF Hellsten U Kawashima T Robinson-Rechavi M Shoguchi E Terry A Yu K Jr Benito-Gutiérrez È Dubchak I Garcia-Fernàndez J Gibson-Brown JJ Grigoriev IV Horton AC De Jong PJ Jurka J Kapitonov VV Kohara Y Kuroki Y Lindquist E Lucas S Osoegawa K Pennacchio LA Salamov AA Satou Y Sauka-Spengler T Schmutz J Shin-I T The amphioxus genome and the evolution of the chordate karyotype. Nature 2008 453 1064 1071 10.1038/nature06967 18563158

[B46] Martin F Kohler A Murat C Balestrini R Coutinho PM Jaillon O Montanini B Morin E Noel B Percudani R Porcel B Rubini A Amicucci A Amselem J Anthouard V Arcioni S Artiguenave F Aury JM Ballario P Bolchi A Brenna A Brun A Buee M Cantarel B Chevalier G Couloux A Da Silva C Denoeud F Duplessis S Ghignone S Perigord black truffle genome uncovers evolutionary origins and mechanisms of symbiosis. Nature 2010 464 1033 1038 10.1038/nature08867 20348908

[B47] Yun SH Arie T Kaneko I Yoder OC Turgeon BG Molecular organization of mating type loci in heterothallic, homothallic, and asexual Gibberella/Fusarium species. Fungal Genet Biol 2000 31 7 20 10.1006/fgbi.2000.1226 11118131

[B48] Fisher MC Hanage WP De Hoog S Johnson E Smith MD White NJ Vanittanakom N Low effective dispersal of asexual genotypes in heterogeneous landscapes by the endemic pathogen penicillium marneffei. PLoS Path 2005 1 0159 0165 10.1371/journal.ppat.0010020 PMC1266309 16254598

[B49] Wittenberg AH van der Lee TA Ben M'barek S Ware SB Goodwin SB Kilian A Visser RG Kema GH Schouten HJ Meiosis drives extraordinary genome plasticity in the haploid fungal plant pathogen *Mycosphaerella graminicola*. PLoS One 2009 4 e5863 10.1371/journal.pone.0005863 19516898 PMC2689623

[B50] Ma LJ van der Does HC Borkovich KA Coleman JJ Daboussi MJ Di Pietro A Dufresne M Freitag M Grabherr M Henrissat B Houterman PM Kang S Shim WB Woloshuk C Xie X Xu JR Antoniw J Baker SE Bluhm BH Breakspear A Brown DW Butchko RA Chapman S Coulson R Coutinho PM Danchin EG Diener A Gale LR Gardiner DM Goff S Comparative genomics reveals mobile pathogenicity chromosomes in *Fusarium*. Nature 2010 464 367 373 10.1038/nature08850 20237561 PMC3048781

[B51] Clutterbuck JA Genomic evidence of repeat-induced point mutation (RIP) in filamentous ascomycetes. Fungal Genet Biol 2011 48 306 326 10.1016/j.fgb.2010.09.002 20854921

[B52] Akagi Y Akamatsu H Otani H Kodama M Horizontal chromosome transfer, a mechanism for the evolution and differentiation of a plant-pathogenic fungus. Eukaryot Cell 2009 8 1732 1738 10.1128/EC.00135-09 19749175 PMC2772402

[B53] Roca M Read N Wheals A Conidial anastomosis tubes in filamentous fungi. FEMS Micro Letts 2005 249 191 198 10.1016/j.femsle.2005.06.048 16040203

[B54] Liu Y Leigh JW Brinkmann H Cushion MT Rodriguez-Ezpeleta N Philippe H Lang BF Phylogenomic analyses support the monophyly of Taphrinomycotina, including *Schizosaccharomyces *fission yeasts. Mol Biol Evol 2009 26 27 34 18922765 10.1093/molbev/msn221 PMC8929177

[B55] Shertz CA Bastidas RJ Li W Heitman J Cardenas ME Conservation, duplication, and loss of the Tor signaling pathway in the fungal kingdom. BMC Genomics 2010 11 510 10.1186/1471-2164-11-510 20863387 PMC2997006

