# Supplementary figures and images for: A novel mode of chromosomal evolution peculiar to filamentous Ascomycete fungi
Source: Genome Biol. 2011 May 24;12(5):R45. doi: 10.1186/gb-2011-12-5-r45 (PMC3219968; doi:10.1186/gb-2011-12-5-r45)

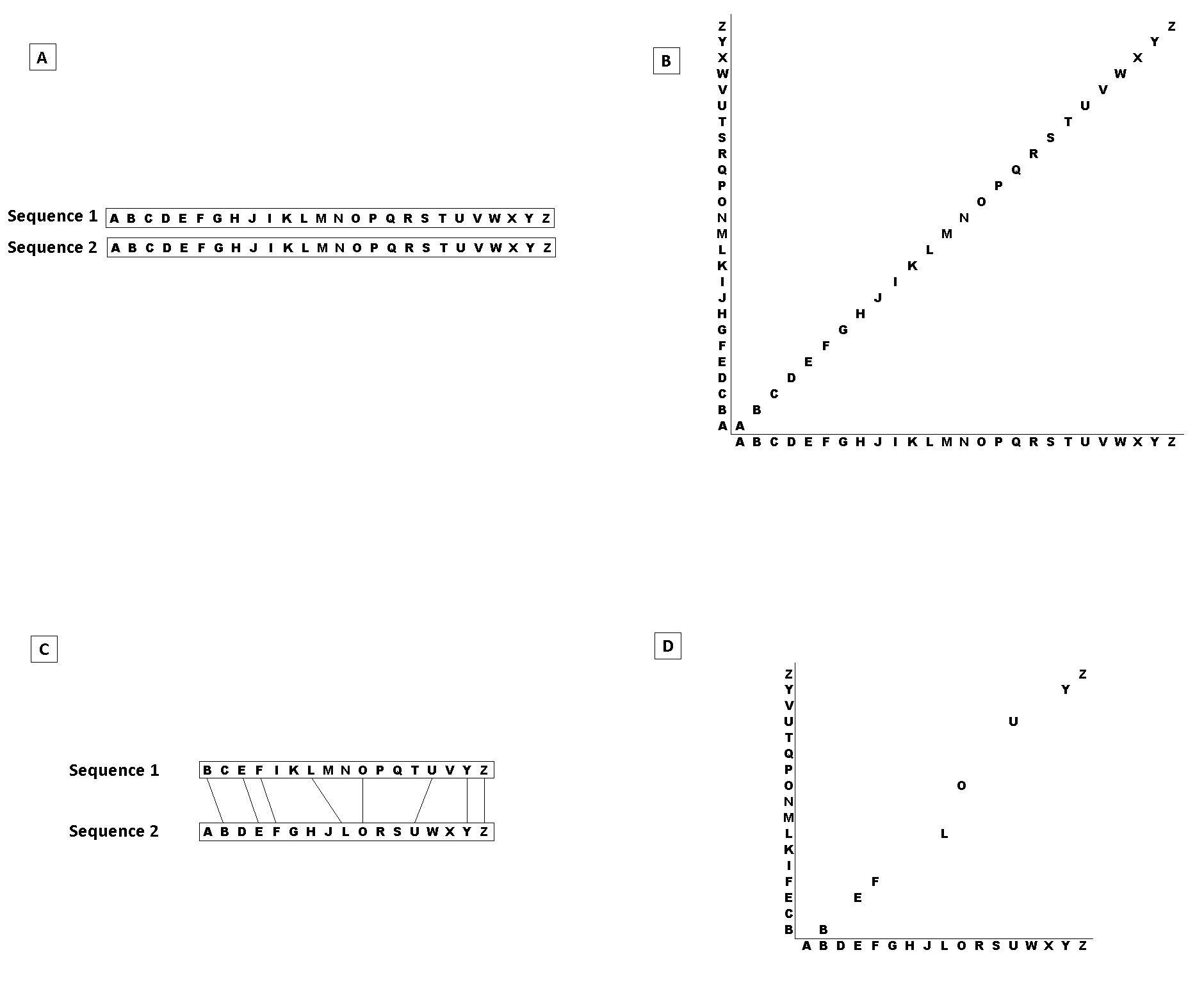

Supplement: Additional file 1 — Supplementary Figure S1. The origins of the different types of syntenic relationships. Immediately after a speciation event, equivalent chromosomes in two daughter species retain the gene content, order and orientation of the parent species. (a) Diagrammatic representation of a chromosome with sequential elements A to Z. (b) A dot plot comparing the chromosomes in (a), with letters substituted for dots. The unbroken series of letters on the diagonal indicates macrosynteny. (c, d) Loss of sequences from each chromosome (c) will degrade the diagonal co-linearity when visualized as a dot plot (d). [file gb-2011-12-5-r45-S1.PNG]

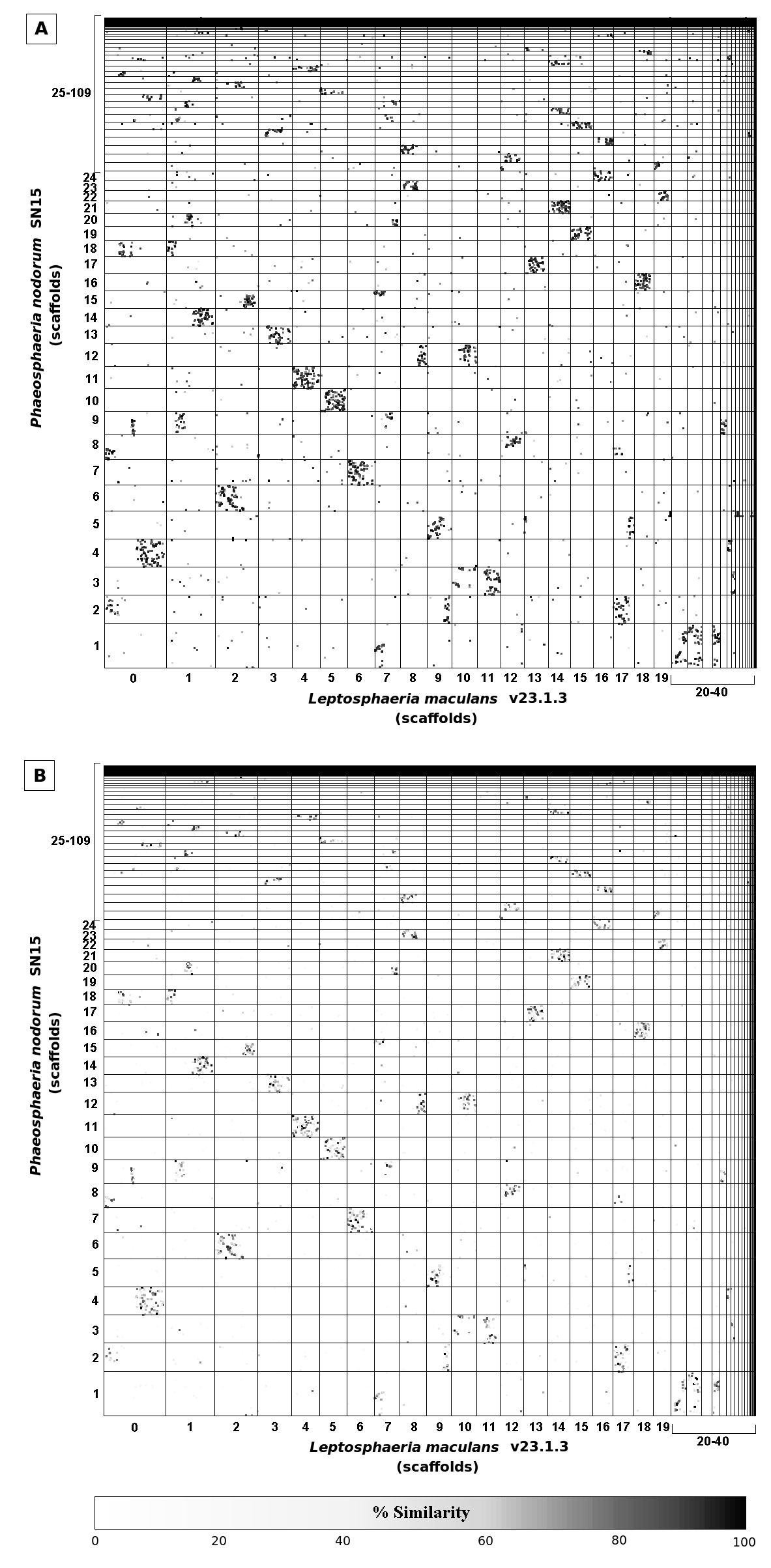

Supplement: Additional file 2 — Supplementary Figure S2. (a, b) Correspondence between promer-derived dot plots (a) and blastp-derived protein comparisons of annotated genes (b) between Phaeosphaeria nodorum and Leptosphaeria maculans. Sequence pairs ('boxes') in (a) containing non-random distributions of 'dots' correspond to those in (b), indicating that the back-translated genome matches in (a) correspond to regions of conserved gene content. [file gb-2011-12-5-r45-S2.PNG]

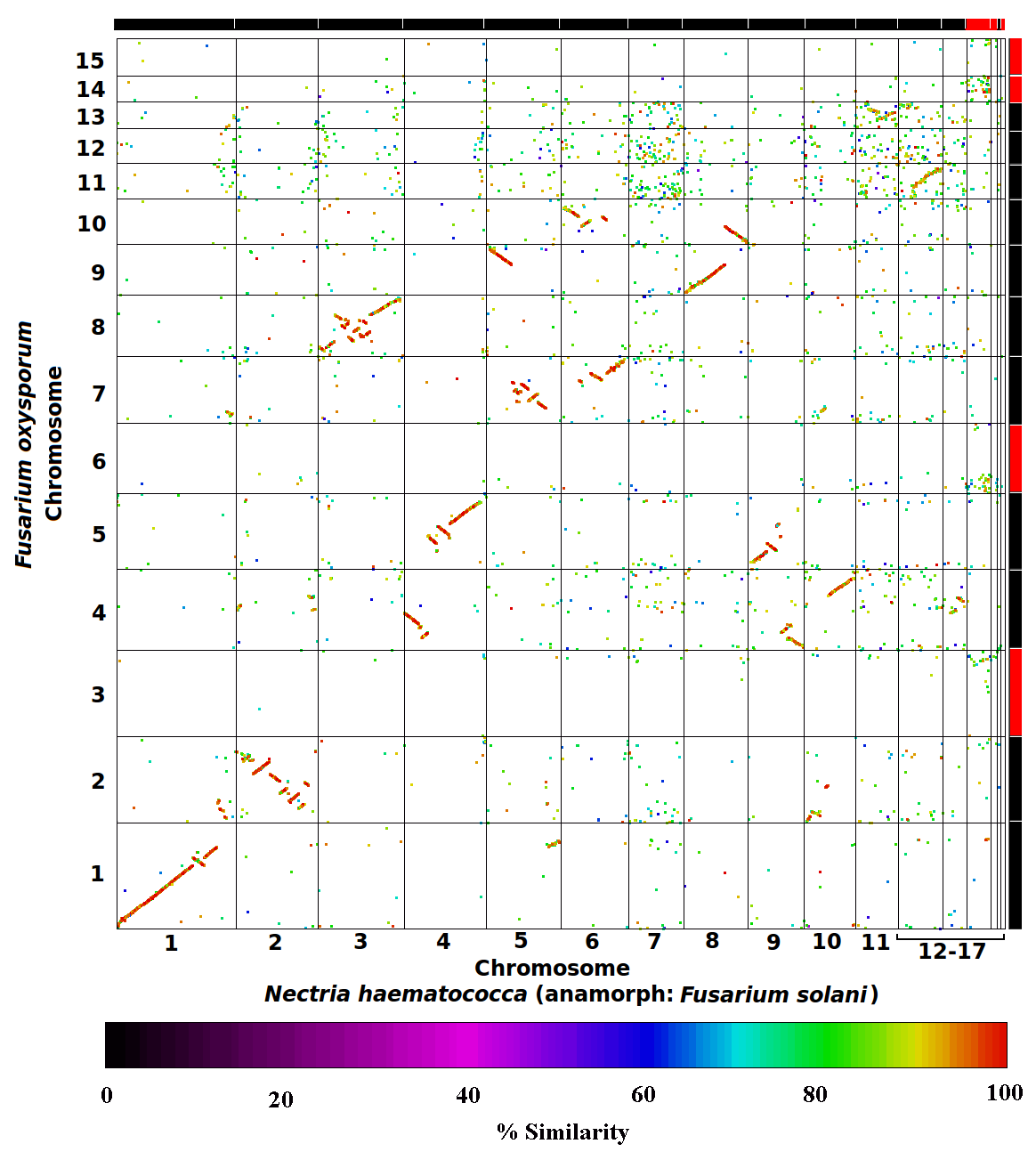

Supplement: Additional file 4 — Supplementary Figure S3. Presence of both macrosyntenic and mesosyntenic conservation patterns between the genomes of Fusarium oxysporum and Nectria haematococca. Core chromosomes (indicated by black bars along the axes) are macrosyntenic between the two species. Dispensable chromosomes (red bars along the axes) are either non-syntenic (N. haematococca chromosomes 15 to 17) or mesosyntenic (N. haematococca chromosomes 7 and 11 to 14, F. oxysporum chromosome 14). The majority of chromosomes 3 and 6 of F. oxysporum had no similarity to the chromosomes of N. haematococca except for regions near their telomeres. [file gb-2011-12-5-r45-S4.PNG]

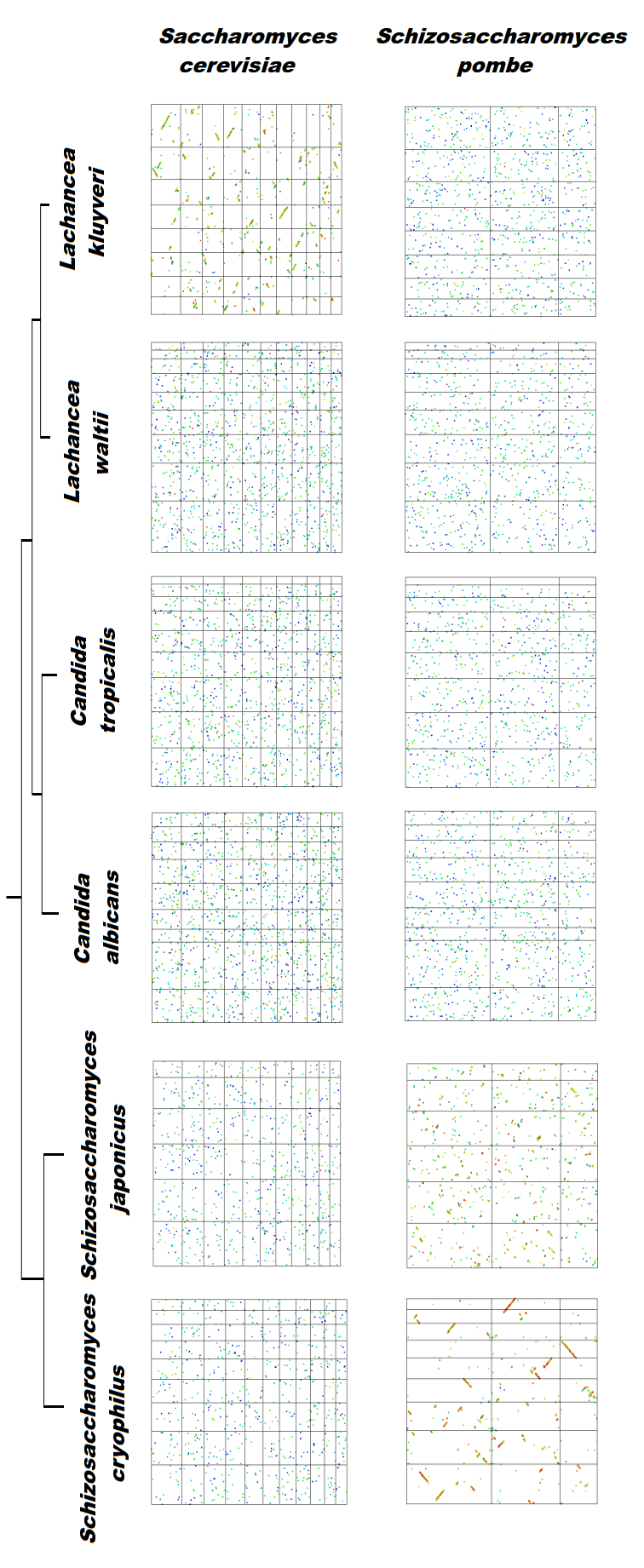

Supplement: Additional file 5 — Supplementary Figure S4. Degradation of whole-genome synteny in the classes Saccharomycetes and Schizosaccharomyces. Whole-genome dot plots have been limited to scaffolds or chromosomes greater than 500 kb. Species of the Saccharomyces and Schizosaccharomyces do not exhibit whole-genome conservation with each other. Certain species within each class exhibit macrosynteny whereas others exhibit no synteny. [file gb-2011-12-5-r45-S5.PNG]

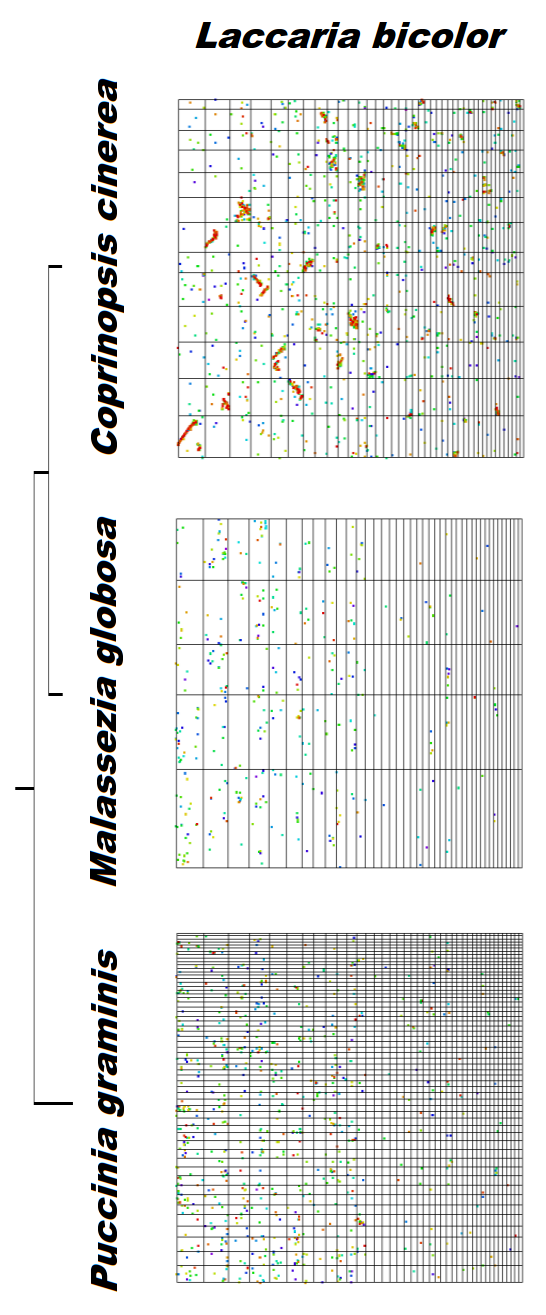

Supplement: Additional file 6 — Supplementary Figure S5. Degradation of whole-genome synteny between a member of the class Agaricales and related orders. Whole-genome dot plots have been limited to scaffolds or chromosomes greater than 500 kb. Species in the Agaricales exhibited macrosynteny with each other. However, the Agaricales exhibited no synteny with the closest related classes represented in this study, the Exobasidiomycetes and Pucciniomycetes. [file gb-2011-12-5-r45-S6.PNG]

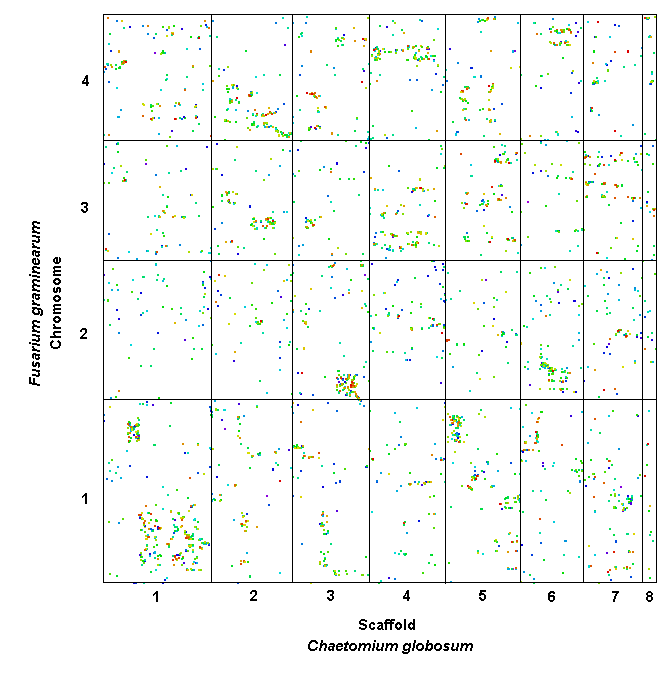

Supplement: Additional file 7 — Supplementary Figure S6. Evidence of degraded mesosynteny between the genomes of two homothallic Sordariomycete species, Fusarium graminearum (order Hypocreales) and Chaetomium globosum (order Sordariales). These two species are estimated to have diverged approximately 225 Mya. Sequence matches (dots) are arranged in blocked clusters typical of mesosynteny. Chromosomes and scaffolds do not share a one-to-one relationship, with multiple mesosyntenic clusters appearing in the same row or column. [file gb-2011-12-5-r45-S7.PNG]
